# Artificial intelligence-based endoscopic ultrasonography model for detecting the origin layer of gastric subepithelial lesions

**DOI:** 10.3389/fmed.2026.1802113

**Published:** 2026-03-12

**Authors:** Xiao Wang, Liangpeng Pu, Shanshan Yan, Shuaishuai Zhuang, Jie Hua, Xiaopu He

**Affiliations:** 1 Department of Geriatric Gastroenterology, The First Affiliated Hospital of Nanjing Medical University, Nanjing, Jiangsu, China; 2 Department of Gastroenterology, The First Affiliated Hospital of Nanjing Medical University, Nanjing, Jiangsu, China

**Keywords:** artificial intelligence, deep learning, endoscopic ultrasonography, gastric subepithelial lesions, MedMamba

## Abstract

**Background:**

Gastric subepithelial lesions (SELs) are typically covered by intact mucosa, which challenges the determination of their layer of origin under conventional endoscopy. Endoscopic ultrasonography (EUS) is indispensable for diagnosing SELs. However, EUS operation and interpretation are more challenging than standard endoscopy and are subject to inter-observer variability. This study aimed to develop a novel AI system based on the MedMamba architecture to assist clinicians in identifying the layer of origin of gastric SELs.

**Methods:**

We retrospectively collected data from patients who underwent EUS at the First Affiliated Hospital of Nanjing Medical University between May 1, 2016 and May 1, 2023. The dataset comprised 1,855 images from 320 patients. Images were split into training, validation, and test sets at an 8:1:1 ratio at the patient level to prevent data leakage. A structured State space sequence model (MedMamba) was trained and the performance was compared against endoscopists.

**Results:**

The proposed MedMamba model achieved an overall accuracy of 92.04% (95%CI: 90.33–93.75%), with 94.83% (95%CI: 93.22–96.44%) specificity and 81.11% (95%CI: 75.19–87.03%) sensitivity in the five-category classification. It achieved high sensitivity and accuracy, outperforming other AI models.

**Conclusion:**

The MedMamba-based AI system demonstrated superior performance in discriminating the layer of origin of SELs compared with other AI models, indicating its potential utility in reducing diagnostic variability and enhancing clinical diagnostic workflows.

## 1 Introduction

Subepithelial lesions (SELs) can originate from any layer of the digestive tract wall. During conventional upper gastrointestinal endoscopy, these lesions typically present as smooth protrusions covered with normal mucosa (1). Typically, these lesions are small and asymptomatic; however, in some cases, they may lead to symptoms such as dysphagia, abdominal pain, overt or occult gastrointestinal bleeding, and luminal obstruction (2). The majority of SELs are benign; however, approximately 20% are malignant (1). The prognostic spectrum is broad, encompassing benign and indolent tumors to malignant and potentially aggressive ones, such as leiomyomas (LMs), gastrointestinal stromal tumors (GISTs), and Borrmann type I advanced gastric cancers (3, 4). LMs and GISTs typically originate from the muscularis propria layer. In contrast, early gastric cancer (which may include the elevated type) can arise from the mucosal layer, and may progress by infiltrating into deeper gastric wall layers (5). It is crucial to clearly differentiate between true subepithelial lesions and early gastric cancer (EGC). Although EGC, particularly elevated types, may invade the submucosal layer, it originates from the mucosal layer and therefore does not belong to the category of true subepithelial (submucosal) lesions. This conceptual distinction is of significant clinical relevance.

Endoscopic ultrasonography (EUS) is the cornerstone modality for evaluating gastric elevated lesions (6). It builds upon white-light endoscopy by delineating lesions infiltrating the gastric wall layers and defining their relationship to surrounding structures. Furthermore, EUS is instrumental in assessing lesion size, morphology, layer of origin, and depth of invasion (7–9). However, the diagnostic accuracy of EUS for discriminating the layer of origin requires improvement, with reported rates ranging from 45.5 to 66.7% (10). The identification of the layer of origin is highly operator-dependent, and diagnostic accuracy is notably lower for lesions smaller than 2 cm (11). While these procedures are invasive and may be associated with adverse events such as bleeding or, rarely, perforation, tissue acquisition remains a key component in the diagnostic work-up of subepithelial lesions, particularly when lesions are of adequate size and technically accessible. Histological assessment is often necessary to guide risk stratification and subsequent management decisions (1, 12, 13). Therefore, a non-invasive, accurate diagnostic support tool is clinically needed to guide management decisions, such as distinguishing lesions requiring resection from those suitable for surveillance.

Recently, deep learning has shown promising results in medical image analysis. The Mamba model, an innovative architecture, integrates classical convolutional layers with state space models (SSMs). Mamba offers linear computational complexity while effectively capturing global contextual features (14–16). This efficiency makes it particularly suitable for clinical deployment where computational resources may be constrained (17–19). To bridge the gap between real-world clinical scenarios and AI applications, it is essential to recognize the sequential diagnostic workflow for SELs. While conventional endoscopy serves as the primary screening tool to detect these lesions, it cannot classify their layer of origin as they are typically covered by intact normal mucosa. Consequently, EUS is employed as the secondary, definitive modality for anatomical classification. Our proposed AI system is specifically designed to seamlessly integrate into this downstream EUS phase, acting as an advanced decision-support tool to automate and enhance the accuracy of layer classification.

The primary objective of this study was to evaluate the diagnostic performance of a MedMamba-based AI system in classifying the layer of origin of gastric SELs. The secondary objective was to compare its performance against expert and non-expert endoscopists to assess its incremental value in reducing inter-observer variability.

## 2 Methods

### 2.1 Study design and ethical considerations

This retrospective study was conducted using EUS images obtained from patients who presented at the Endoscopy Center of the First Affiliated Hospital of Nanjing Medical University between May 1, 2016 and May 1, 2023. This retrospective study involving human participants was approved by the Ethics Committee of the First Affiliated Hospital of Nanjing Medical University (IRB No. 2025-SR-947). The requirement for written informed consent was waived due to the retrospective design and use of de-identified data. This study followed the TRIPOD-AI reporting guidelines for prediction model development and validation. A completed checklist is provided in the Supplementary material.

### 2.2 EUS equipment and dataset preparation

EUS examinations were performed by expert endoscopists using equipment from Olympus (TJF-260V, TJF-240, GIF-Q260, GIF-Q260J, GIF-H260, GF-UE260) and Fujifilm (EG29-I10, EG-600UR), Japan. To handle potential domain shifts caused by different devices, images from all available processors were included in the training process.

**Figure 1:**
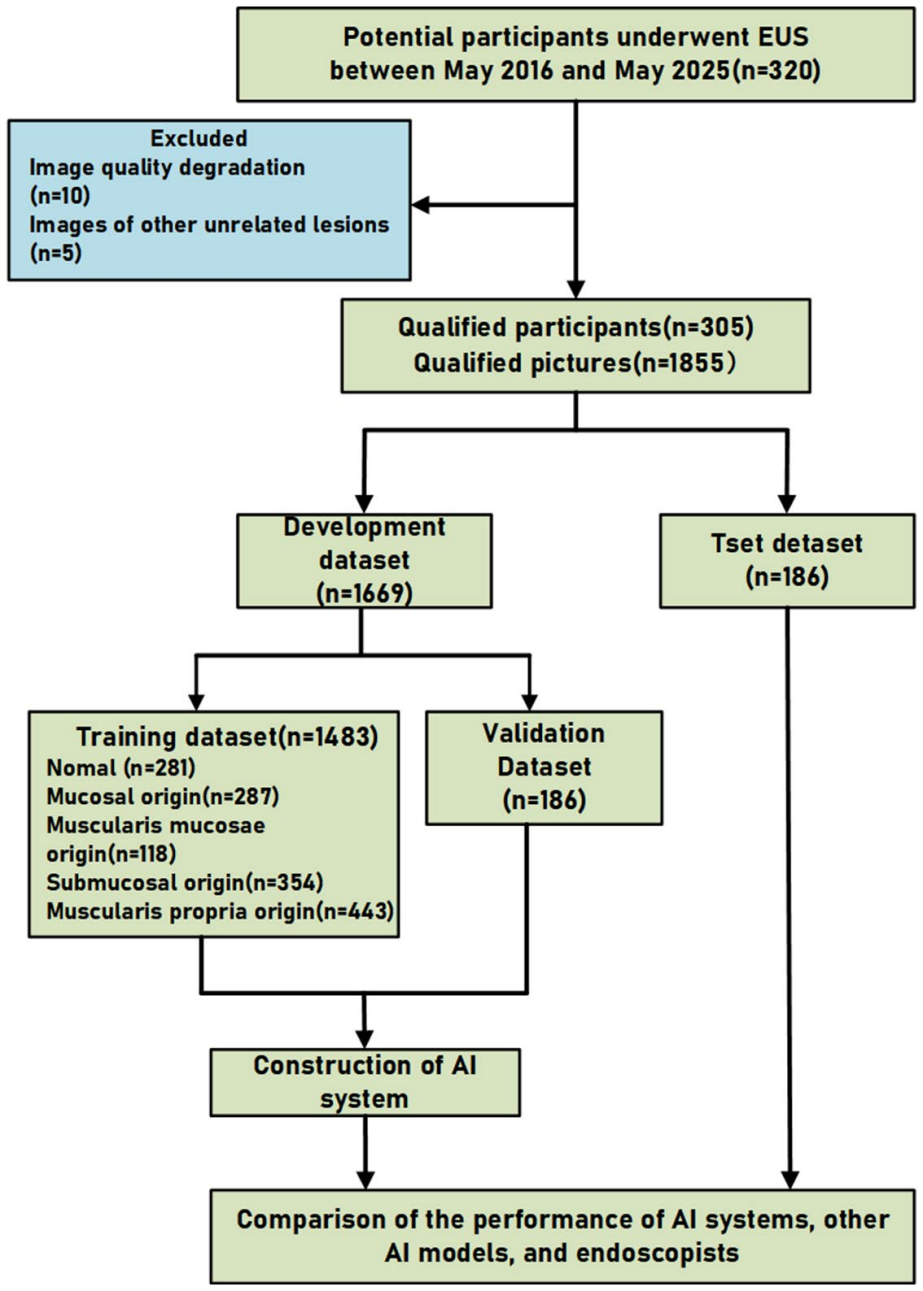
Flowchart of the study.

The layer of origin for all lesions was diagnosed by certified endoscopic experts in accordance with the American Society for Gastrointestinal Endoscopy (ASGE) guidelines, integrating findings from conventional endoscopy, EUS, and available follow-up data. While histopathology served as the gold standard where resection or biopsy was performed, for cases under surveillance, the consensus EUS diagnosis by senior experts served as the reference standard. Histopathological confirmation was obtained in 21% of the cases through either EUS-guided fine-needle aspiration (EUS-FNA) or endoscopic resection. For the remaining 79% of cases, primarily small (<2 cm) and asymptomatic lesions exhibiting typical benign sonographic features, the diagnosis was established based on the consensus of senior endoscopists and confirmed by stable follow-up findings, in adherence to current clinical guidelines where invasive tissue sampling was not indicated. 320 patients were initially identified. Among them, ten patients were excluded due to poor EUS image quality, and a further five were excluded as their images were unrelated to the study focus. Consequently, 305 patients were included in the final analysis (1,855 EUS images). To ensure robust evaluation and prevent data leakage, the dataset was split into training, validation, and test sets at an 8:1:1 ratio at the patient level. The selection process is provided in Figure 1.

### 2.3 Image annotation and augmentation

**Figure 2:**
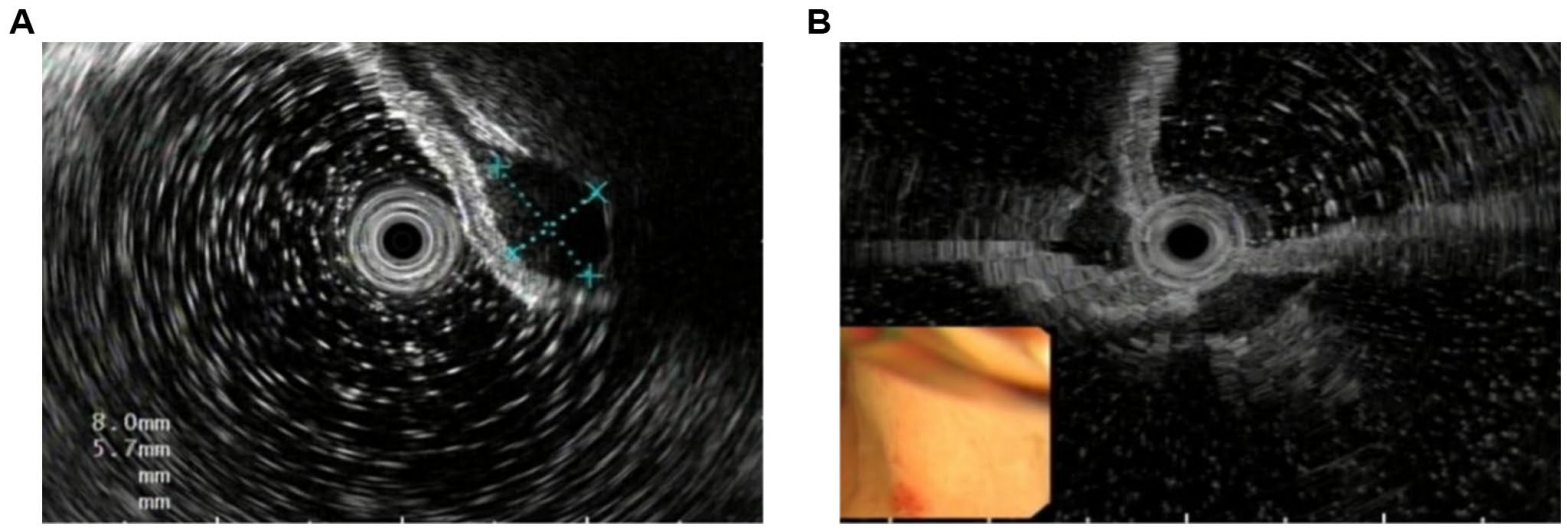
Eligible and ineligible image examples **(A)** eligible image. **(B)** ineligible image.

Images were classified according to the confirmed layer of lesion origin (mucosa, muscularis mucosae, submucosa, or muscularis propria). Ineligible images were excluded based on the following criteria: (i) compromised image quality (e.g., due to blurring, noise, obscuring mucus, bubbles, or food residue); (ii) presence of lesions unrelated to the study focus; and (iii) duplicate images. Figure 2 shows the difference between clear and blurred images. The lesions were then delineated as the regions of interest (ROI) by junior doctors using LabelMe (MIT Computer Science and Artificial Intelligence Laboratory, Cambridge, MA, USA), an open-source annotation tool developed by the MIT Computer Science and Artificial Intelligence Laboratory. Subsequently, images were cropped to square or rectangular formats based on tight-fitting bounding boxes around the ROIs to minimize background interference. To improve model generalization and robustness, standard data augmentation techniques were applied to the training set, including random rotation, flipping, and brightness adjustment. All annotations and cropped images were reviewed and verified by senior endoscopists. During the annotation process, physicians considered key sonographic features of the lesions, including echogenicity and border definition, which are indicative of the layer of origin. Any discrepancies in annotation were resolved through consensus discussion among the senior endoscopists to ensure annotation accuracy.

### 2.4 AI model construction and performance evaluation

#### 2.4.1 MedMamba

**Figure 3:**
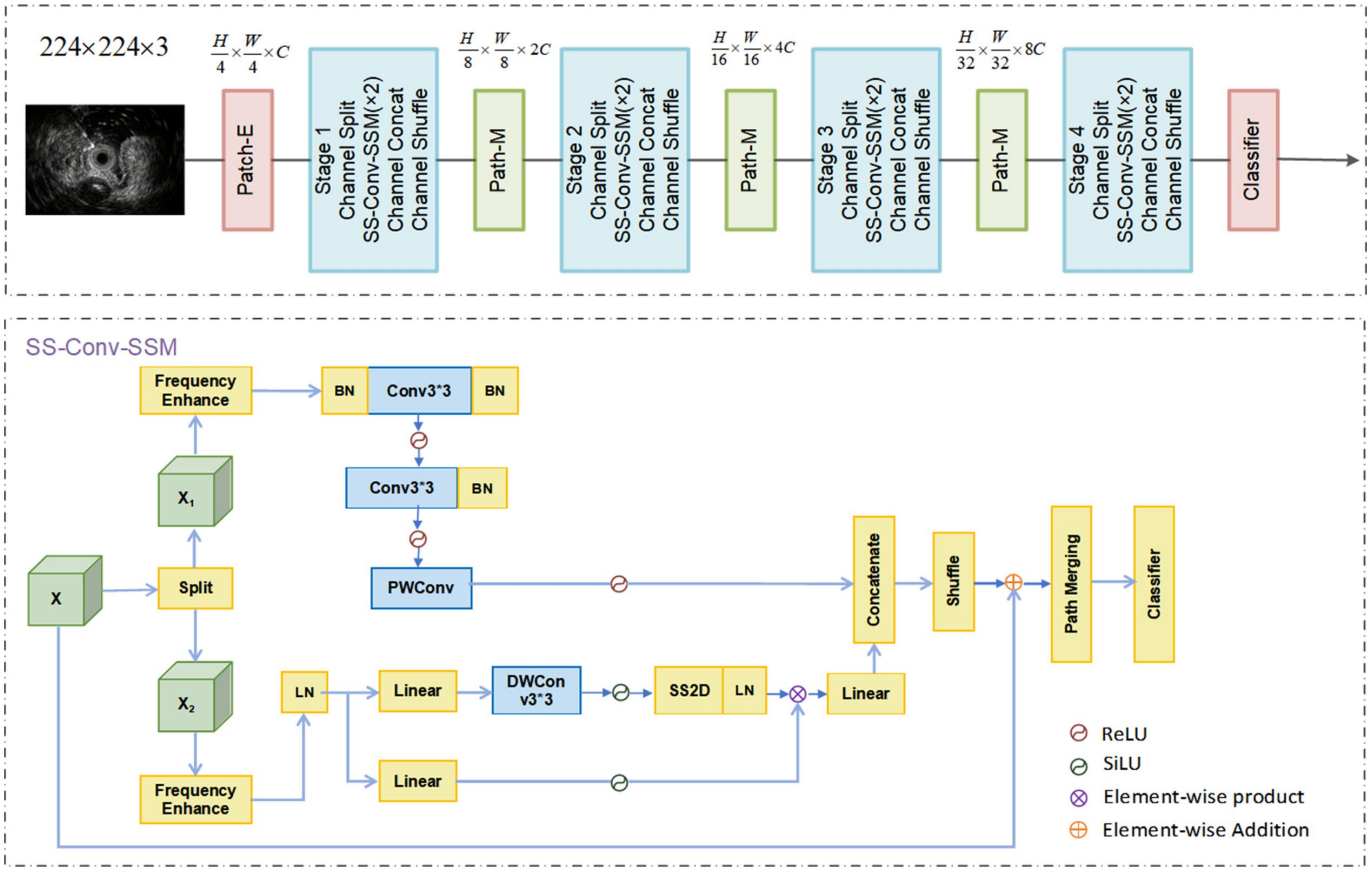
Overall architecture of MedMamba. Patch-E and Patch-M represent patch-embedding and patch-merge, respectively. BN, LN, Linear, PWConv, and DWConv represent batch normalization, layer normalization, linear layer, pointwise convolution, and depthwise separable convolution, respectively.

In this study, we constructed the AI system based on the MedMamba architecture, which leverages state space models (SSMs) and a discretization process (14, 15, 20). The architecture consists of a patch embedding layer, serially stacked SS-Conv-SSM blocks, patch merging layers for down-sampling, and a classification head. Similar to Vision Transformers (ViTs), MedMamba first partitions the input image into patches (16, 21). Unlike standard ViTs, it does not flatten these patches into a 1D sequence. The patch embedding layer produces a feature map of dimensions H × W × C, thereby preserving the 2D spatial structure of the input image. This feature map is then processed by Stage-1, which consists of repeatedly stacked SS-Conv-SSM blocks that operate without altering the feature dimensions. To construct a hierarchical feature representation, MedMamba employs patch merging layers to downsample the feature maps between stages. This process is repeated through Stage-2, Stage-3, and Stage-4. Subsequent patch merging layers downsample the output of Stage-2 to a resolution of $\frac{H}{16}\times \frac{H}{16}\times 4C$ and Stage-3 to $\frac{H}{32}\times \frac{H}{32}\times 8C$ . Finally, the classification head, comprising an adaptive global average pooling layer followed by a linear layer, computes the class probabilities for the input image. The overall data flow in MedMamba follows an architecture similar to hierarchical CNNs and ViTs. The input image size was set to 224 × 224 pixels with 3 color channels. The overall architecture of MedMamba is illustrated in Figure 3.

The 2D selective scan (SS2D) module, a core component of MedMamba, was originally proposed by VMamba (22). SS2D adapts the selective scan state space model (S6) from natural language processing to visual data, effectively resolving the inherent “direction-sensitive” limitation of S6 in this domain. To bridge the gap between 1D sequential scanning and 2D spatial traversal, SS2D introduces the Cross-Scan Module (CSM) (23). This module enables the effective extension of S6 to visual data while preserving a global receptive field. The CSM employs a four-directional scanning strategy, traversing the feature map from each of its four corners towards the opposite edge. This strategy ensures that each pixel integrates contextual information from all other pixels across different directions, thereby establishing a global receptive field while maintaining linear computational complexity.

By integrating the CSM, SS2D retains the linear computational complexity of S6 while conferring the ability to capture long-range dependencies—a critical property for accurate medical image classification. The S6 block, integral to the Mamba architecture, introduces a selection mechanism by dynamically parameterizing the SSM based on the input, evolving from the static S4 model. This dynamic parameterization enables the model to selectively retain relevant information and filter out irrelevant cues.

#### 2.4.2 Frequency domain enhancement method

To further augment the model’s representational capacity, we incorporated a frequency domain enhancement method. In image processing, low-frequency components typically encapsulate structural information, whereas high-frequency components capture fine-grained details. To optimize the model for clinical applicability rather than algorithmic complexity, we utilized a frequency domain enhancement method. In EUS imaging, low-frequency components typically represent the overall structure of the gastrointestinal wall, whereas high-frequency components capture fine details such as borders and internal echogenicity. By integrating these features, the model is designed to better mimic the clinical evaluation of subtle ultrasonographic variations between adjacent anatomical layers, thereby improving the practical implications of the model for endosonographers.

#### 2.4.3 Model training and outcome measures

All models were implemented and trained using the PyTorch deep learning framework. The initial learning rate was set to 0.001 and decayed exponentially by a factor of 0.9 every 10 epochs. Optimization was performed using the Adam algorithm with its default momentum parameters (*β*₁ = 0.9, *β*₂ = 0.999). The model was trained on the training set, with the validation set used for hyperparameter tuning and early stopping. The held-out test set was used exclusively for the final performance evaluation.

The primary outcome was the diagnostic performance of the AI system in classifying the layer of origin. Secondary outcomes included a comparative analysis of the diagnostic performance between the AI system, endoscopists (experts and non-experts), and other benchmark deep learning models. Expert endoscopists were defined as those with over 10 years of experience in evaluating gastrointestinal lesions and having performed more than 2000 EUS procedures. Non-expert endoscopists had 1–2 years of experience and had performed fewer than 300 EUS procedures.

### 2.5 Statistical analysis

All statistical analyses were conducted using SPSS (Version 26.0; IBM Corp., Armonk, NY, USA) and MedCalc Statistical Software (Version 15.0; MedCalc Software Ltd., Ostend, Belgium). Diagnostic performance was evaluated using Area Under the Receiver Operating Characteristic Curve (AUC), sensitivity (SEN), specificity (SPE), positive predictive value (PPV), negative predictive value (NPV), and accuracy (ACC). 95% confidence intervals (95CIs) were calculated for key metrics to assess the precision of the estimates. Differences between the AI system and endoscopists were compared using the two-sided McNemar’s test. Continuous variables were presented as median and range. Categorical variables are summarized using frequencies and percentages. To evaluate the clinical interpretability of the proposed model, a representative feature activation heatmap was generated. This visualization aims to highlight the spatial regions within the EUS images that most significantly influence the model’s classification decisions, thereby verifying that the model focuses on the lesion rather than background artifacts.

## 3 Results

### 3.1 Basic information

The analysis included 305 patients, comprising 130 males (42.6%) and 175 females (57.4%). The age of the patients ranged from 19 to 81 years [Mean age: 55.6 ± 12.1 years (mean ± SD)], the majority were in the 50–69 age group. Table 1 details the distribution of cases.

**Table 1:** Statistics distribution from gastric lesion database.

Dataset	Normal	Mucosal origin	Muscularis mucosae origin	Submucosal origin	Muscularis propria origin
Train	10	28	10	65	101
Validation	2	6	3	13	22
Test	2	6	2	13	22
Total	14	40	15	91	145

### 3.2 Model interpretability

**Figure 4:**
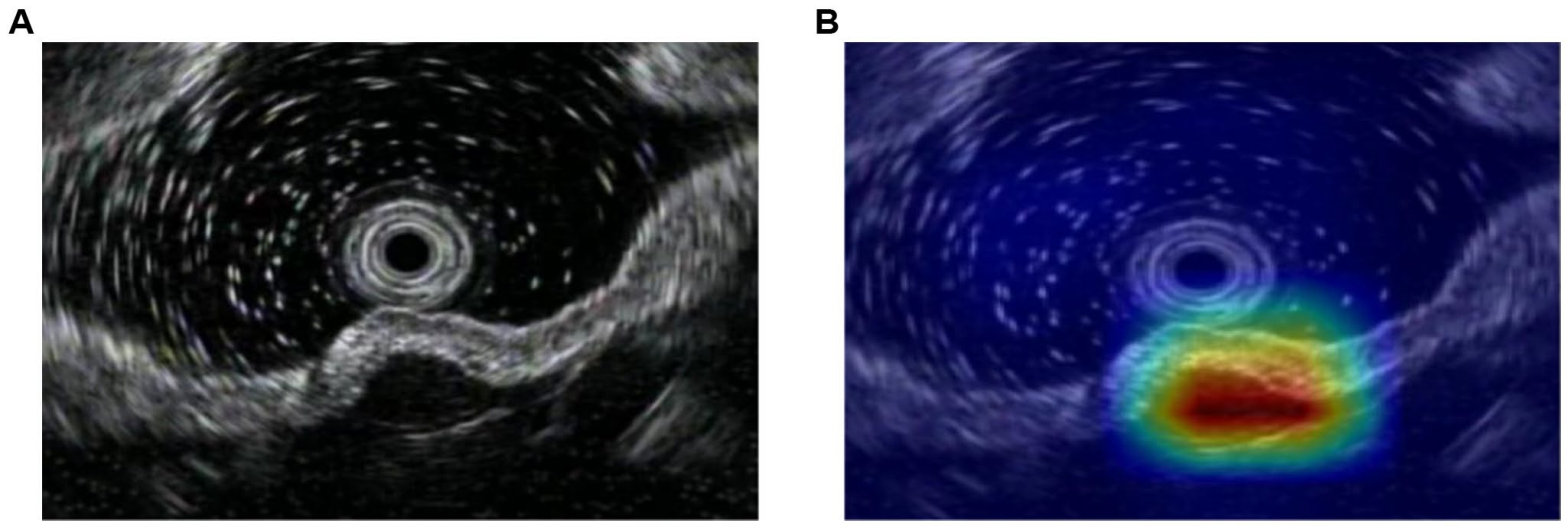
Visualization of model interpretability. **(A)** A representative original endoscopic ultrasonography (EUS) image showing a gastric subepithelial lesion. **(B)** The corresponding feature activation heatmap generated by the MedMamba model. Warmer colors (red) indicate regions with higher influence on the model’s classification decision, demonstrating that the model accurately focuses on the target lesion rather than surrounding artifacts or healthy tissue. **(A)** Original image, **(B)** Heatmap of the leision.

To ensure that the model bases its predictions on clinically relevant anatomical features rather than background noise, we visualized the model’s spatial focus. Figure 4 presents a representative original EUS image alongside its corresponding feature activation heatmap. The visualization demonstrates that the highest activation regions (indicated by warmer colors) closely align with the target gastric subepithelial lesion, suggesting that the model focuses on lesion regions.

### 3.3 Performance of the proposed model

The model achieved an overall accuracy of 92.04% (95% CI: 90.33–93.75%). For lesions originating from the mucosa, muscularis mucosae, submucosa, and muscularis propria, the accuracies were 91.40% (95% CI: 87.37–95.43%), 95.70% (95% CI: 92.79–98.61%), 87.63% (95% CI: 82.90–92.36%), and 86.56% (95% CI: 81.66–91.46%) respectively. The relatively lower accuracy observed for lesions originating from the submucosa and muscularis propria may be attributed to the increased complexity and potential similarity of sonographic features in deeper gastric wall layers. The classification performance of the MedMamba model is detailed in Table 2.

**Table 2:** Results of MedMamba in gastric lesion database (95%CI).

Class	Accuracy	Sensitivity	Specificity	PPV	NPV
Normal	98.92% (97.43–100.00%)	100.00% (90.11–100.00%)	98.68% (96.86–100.00%)	94.59% (87.30–100.00%)	100.00% (97.49–100.00%)
Mucosal	91.40% (87.37–95.43%)	83.33% (71.16–95.50%)	93.33% (89.34–97.32%)	75.00% (61.58–88.42%)	95.89% (92.67–99.11%)
Muscularis mucosae	95.70% (92.79–98.61%)	80.00% (59.76–100.00%)	97.08% (94.56–99.60%)	70.59% (48.93–92.25%)	98.22% (96.23–100.00%)
Submucosal	87.63% (82.90–92.36%)	63.64% (49.43–77.85%)	95.07% (91.51–98.63%)	80.00% (66.75–93.25%)	89.40% (84.49–94.31%)
Muscularis propria	86.56% (81.66–91.46%)	78.57% (67.82–89.32%)	90.00% (84.84–95.16%)	77.19% (66.30–88.08%)	90.70% (85.69–95.71%)
Average	92.04% (90.33–93.75%)	81.11% (75.19–87.03%)	94.83% (93.22–96.44%)	79.47% (73.16–85.78%)	94.84% (91.67–98.02%)

### 3.4 Comparative diagnostic performance

We compared the diagnostic performance of MedMamba against 7 other AI models. All the models were evaluated on the entire test set. The results demonstrated that MedMamba achieved high diagnostic performance in diagnosing the layer of origin. It attained the highest scores in Accuracy [92.04% (95%CI: 90.33–93.75%)], Recall [81.11% (95%CI: 75.19–87.03%)], and F1-score (0.799) among all compared models. The performance of MedMamba can be ascribed to its unique architecture that synergistically combines convolutional layers for local feature extraction with state space models for capturing long-range dependencies. Furthermore, the novel SS-Conv-SSM block enhances feature extraction efficiency through the integration of channel splitting, convolutional layers, SSM layers, and channel shuffle operations, which is particularly beneficial for interpreting complex EUS image patterns. The comprehensive diagnostic performance metrics of MedMamba and others are presented in Table 3.

**Table 3:** Performance of MedMamba and other AI models (95%CI).

Method	Accuracy	Precision	Recall	F1-score
MedMamba	92.04% (90.33–93.75%)	79.47% (73.16–85.78%)	81.11% (75.19–87.03%)	0.799
ResNet-101	68.91% (67.67–70.02%)	88.40% (83.98–92.77%)	31.42% (25.53–37.91%)	0.261
ResNet-50	69.53% (66.35–72.87%)	88.42% (84.12–93.75%)	30.65% (24.01–27.88%)	0.247
VGG-16	65.64% (63.68–68.55%)	87.22% (82.34–92.12%)	29.31% (23.35–35.12%)	0.226
ViT	61.60% (59.31–62.03%)	88.42% (82.78–93.63%)	29.32% (22.88–35.79%)	0.222
ViT + TS	76.33% (73.98–78.82%)	90.56% (85.44–95.57%)	39.76% (33.65–45.29%)	0.347
ViT + TS + RR	77.45% (74.12–80.01%)	92.33% (87.99–98.23%)	35.32% (29.87–42.55%)	0.313
SRRM-ViT	78.77% (76.22–80.53%)	92.12% (86.63–98.87%)	41.12% (36.90–47.15%)	0.368

**Figure 5:**
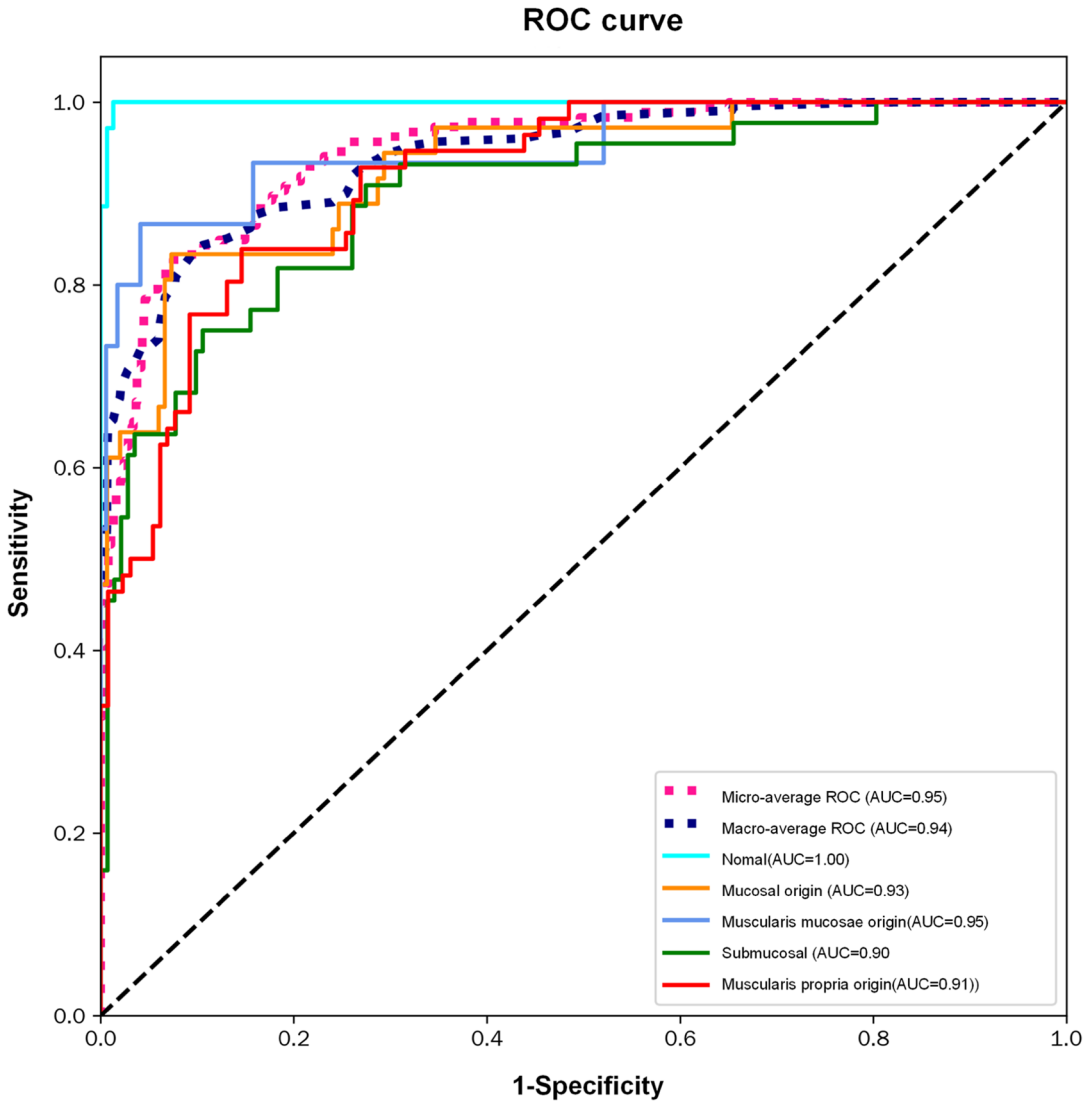
ROC curves.

To evaluate the diagnostic robustness of the MedMamba system, Receiver Operating Characteristic (ROC) curves were generated (Figure 5). The quantitative analysis demonstrated that MedMamba achieved a macro-average AUC of 0.94 and a micro-average AUC of 0.95, reflecting high discriminative consistency across various origin layers. Specifically, the model exhibited optimal performance in identifying mucosal origin (AUC 0.95), while maintaining substantial accuracy for deeper structures like the submucosa (AUC 0.90) and muscularis propria (AUC 0.91). Comparative evaluations indicated that MedMamba significantly outperformed benchmark models, including ResNet-101 (Accuracy: 92.04% vs. 68.91%) and standard ViT (Recall: 81.11% vs. 29.32%), demonstrated higher performance than the models evaluated in this study. This superior performance can be attributed to the architectural synergy between convolutional layers and State Space Models (SSMs). Unlike traditional CNNs that may overlook long-range spatial dependencies or Transformers that suffer from quadratic computational complexity, the MedMamba’s SS-Conv-SSM block facilitates the simultaneous extraction of localized textures and global contextual relationships. Furthermore, the integration of the 2D Selective Scan (SS2D) module and Cross-Scan Module (CSM) effectively mitigates directional sensitivity in EUS images, ensuring a comprehensive receptive field that is critical for distinguishing subtle echogenic variations between adjacent gastric wall layers.

**Figure 6:**
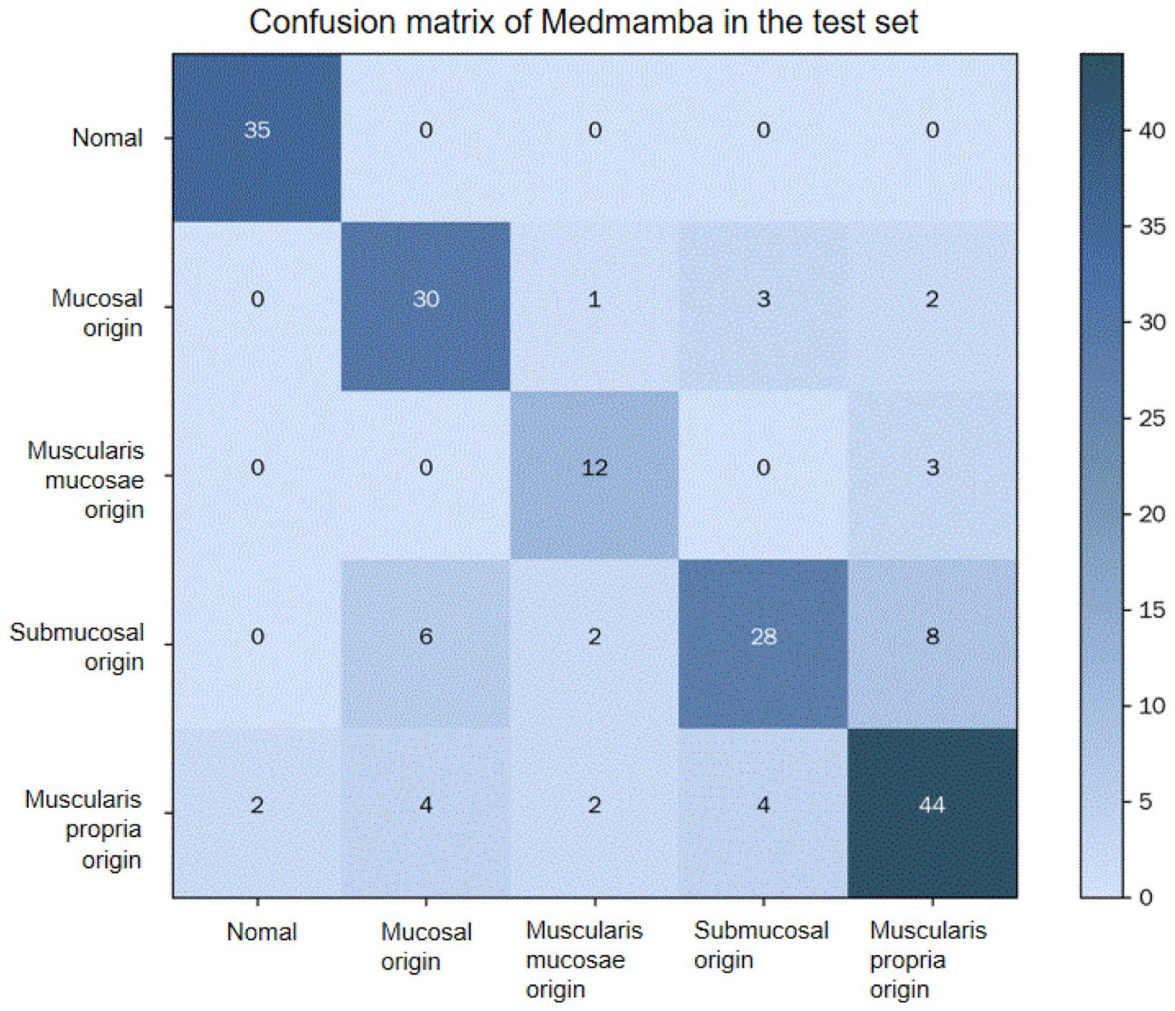
Confusion matrix.

Figure 6 presents the confusion matrix of the proposed method. A confusion matrix is a tabular visualization used to evaluate the performance of a classification model. In this matrix, each row corresponds to the instances of an actual class, while each column corresponds to the instances of a predicted class. The diagonal entries indicate the number of correctly classified instances for each class, whereas the off-diagonal entries represent the misclassifications, detailing the confusion between specific class pairs. The presented confusion matrix elucidates the performance of the MedMamba model in classifying lesions of different origins. The model demonstrated perfect performance in identifying normal images. However, it exhibited some confusion in distinguishing between lesions originating from the submucosa and the muscularis propria. This challenge is likely attributable to the sonographic feature overlap between these lesion types in EUS images.

## 4 Discussion

Subepithelial lesions (SELs) comprise a heterogeneous group of both benign and potentially malignant conditions (1, 2). While the majority of these lesions are benign, approximately 20% of upper gastrointestinal subepithelial lesions possess malignant potential (1). SELs may develop anywhere in the upper gastrointestinal tract, with the stomach being the most frequent site. They are typically asymptomatic and often discovered incidentally during endoscopic or radiologic examinations. Due to their subepithelial location, standard mucosal biopsies frequently yield nondiagnostic results, rendering the diagnostic process challenging. Accurate diagnosis often benefits from a multimodal approach integrating characteristic imaging features, white-light endoscopic and EUS findings, and, when indicated, EUS-guided tissue acquisition (24, 25). Treatment strategies are determined based on the suspected pathology, lesion size, and anatomic location, ranging from endoscopic resection techniques—such as endoscopic mucosal resection (EMR), endoscopic submucosal dissection (ESD), endoscopic submucosal excavation (ESE), endoscopic full-thickness resection (EFR), and submucosal tunneling endoscopic resection (STER)—to surgical resection in selected cases (1, 4, 26). Histologic confirmation is particularly helpful for larger lesions suspicious for malignancy, such as GISTs, to guide appropriate management. In summary, the management of upper GI subepithelial lesions is complex and necessitates an integrated application of various diagnostic modalities and therapeutic strategies (25, 26). Consequently, enhancing early detection and accurate diagnosis remains a critical goal, as it may facilitate timely intervention, improve clinical outcomes, and potentially contribute to improved diagnostic workflows.

The endoscopic evaluation of subepithelial lesions typically begins with standard white-light endoscopy for initial detection. When suspicious lesions are identified, advanced imaging techniques, including endoscopic ultrasonography (EUS), chromoendoscopy, and magnifying endoscopy, are often employed as complementary diagnostic modalities (25). Among these, EUS, complemented by histopathological analysis, is widely considered a primary modality for the evaluation of upper gastrointestinal subepithelial lesions (7, 26). EUS provides valuable capabilities for direct visualization, allowing for a detailed assessment of lesion size, layer of origin, extent of infiltration, echogenic characteristics, and spatial relationships with surrounding structures (27). Specifically, in gastric lesions, EUS typically delineates the five-layer structure of the gastric wall, assisting in the identification of the specific layer from which intramural lesions originate through the detection of architectural disruptions or alterations (26, 27). This detailed characterization aids in estimating the lesion’s origin, depth of invasion, and relationship to adjacent anatomical structures. Consequently, the application of EUS for evaluating digestive tract wall lesions assists in appropriate risk stratification and therapeutic planning, thereby facilitating early management that may help reduce disease progression.

In recent years, artificial intelligence (AI) has gained significant traction in endoscopic image analysis, contributing to meaningful advancements in diagnostic workflows (28). Within this domain, AI algorithms typically perform hierarchical feature extraction from lesion images, subsequently integrating these multi-level features to support decision-making across various tasks, including lesion classification, recognition, and boundary delineation (29). Most recently, the Mamba architecture has emerged as a promising approach for medical computer vision tasks such as classification, segmentation, and detection (14). This model has shown potential in capturing both local details and global contextual information within medical images, which may enhance its discriminative power for diagnostic assistance.

The present study indicates that the MedMamba model achieved encouraging accuracy in evaluating gastric elevated lesions within this dataset. This study presents an initial application of the MedMamba architecture for estimating the layer of origin of gastric SELs. We developed and validated this system on a dataset of 1,855 EUS images, observing favorable classification performance. The proposed model consistently exhibited competitive performance compared to other AI models in static-image evaluation, suggesting its ability to bridge diagnostic gaps across varying levels of clinical experience. These findings support the potential robustness of the model in discriminating the lesion origin and its attainment of the target classification performance. Collectively, our results highlight the feasibility of integrating AI-based tools like MedMamba into clinical workflows for the stratified analysis of complex medical images.

The architectural design underlying MedMamba contributed significantly to the performance of our deep learning model. Specifically, we implemented MedMamba, a hybrid architecture built upon the SS-Conv-SSM block, which integrates classical convolutional layers with state space models (SSMs) (30–32). This design facilitates the simultaneous extraction of both localized features and long-range contextual dependencies from medical images. The architecture further incorporates grouped convolution and channel shuffle operations, which help reduce model parameters and computational complexity while striving to maintain diagnostic accuracy. A core component of MedMamba is the SS2D module, which helps address the directional sensitivity limitation in visual data processing through its Cross-Scan Module (CSM) (33). This mechanism promotes comprehensive information integration from multiple spatial directions, providing the model with a broader receptive field. The SS-Conv-SSM block operates as a lightweight dual-pathway structure, partitioning feature maps into two groups processed, respectively, by convolutional branches (for local features) and SSM branches (for global dependencies). The subsequent channel concatenation and shuffle operations restore the original channel dimensionality while facilitating cross-group information exchange (34, 35).

We acknowledge several limitations in the present study. First, from a clinical and methodological standpoint, the clinical utility of the proposed AI model should be interpreted with caution. In routine practice, a definitive diagnosis of subepithelial lesions cannot be established solely on the basis of layer identification. Without histological confirmation, identifying the layer of origin serves primarily as an adjunctive anatomical guide. Furthermore, the validation of our analysis is constrained by the absence of a uniform histological reference standard, as the study relied largely on expert interpretation of EUS images. Future studies incorporating definitive histological validation will be necessary to ascertain whether the AI-identified layer consistently aligns with final pathological findings. Second, the use of static EUS images evaluated by expert endoscopists represents a notable limitation. One of the principal advantages of EUS lies in its dynamic, real-time assessment capabilities, including the modification of probe position, water instillation, use of antispasmodic agents, and evaluation of lesion mobility and compressibility. Static image analysis does not fully capture real-life EUS practice and may therefore underrepresent expert diagnostic performance. Consequently, while the AI system demonstrated performance comparable to that of experts on static images, it is highly probable that expert accuracy under real-world dynamic conditions would be superior. Third, the research was a single-center retrospective investigation with a limited sample size. This design carries an inherent risk of selection bias and may constrain the generalizability of our findings across diverse populations. Additionally, the dataset exhibits class imbalance, particularly for lesions originating from the submucosa—a reflection of their lower clinical incidence. This imbalance may affect the model’s performance for underrepresented categories. Future research should prioritize validating and refining this model through prospective, multi-center studies with larger, more balanced datasets. Fourth, the MedMamba model is designed as a downstream classification network, which currently relies on bounding boxes (ROIs) to mitigate background interference from full EUS images. When tested on original, unannotated full-frame images, the classification performance understandably decreases due to exposure to irrelevant anatomical structures and ultrasound artifacts. For future autonomous deployment, we plan to explore the integration of an upstream object detection module (such as YOLO) to automatically identify and crop the lesion. Additionally, detailed patient-level clinical data, such as exact lesion size, location, and symptom status, were not systematically incorporated. Relying solely on image features without clinical metadata limits the comprehensive diagnostic capability of the current system. Integrating such clinical variables with EUS images via a multi-modal learning approach could potentially yield further improvements in diagnostic accuracy. Finally, while we have provided representative heatmaps suggesting that the model focuses on the target lesion, comprehensive visual interpretability for State Space Models remains technically challenging compared to traditional Convolutional Neural Networks (CNNs). Developing specialized, high-resolution attention-mapping techniques for SSM-based architectures remains an important objective for our future work to enhance clinical explainability.

The developed AI system represents a promising adjunctive tool for estimating the layer of origin in subepithelial lesions from EUS images, which may hold value in supporting less experienced endoscopists, particularly in resource-limited settings.

## Data Availability

The raw data supporting the conclusions of this article will be made available by the authors, without undue reservation.
